# Dynamic Factor Analysis for Multivariate Time Series: An Application to Cognitive Trajectories

**DOI:** 10.23937/2469-5831/1510001

**Published:** 2015-08-28

**Authors:** Yorghos Tripodis, Nikolaos Zirogiannis

**Affiliations:** 1 Department of Biostatistics, Boston University, USA; 2 School of Public and Environmental Affairs, Indiana University, USA

**Keywords:** Dynamic factor models, EM algorithm, Panel data, State-space models, Cognition, Alzheimer’s disease, Neuropsychological performance

## Abstract

We propose a dynamic factor model appropriate for large epidemiological studies and develop an estimation algorithm which can handle datasets with large number of subjects and short temporal information. The algorithm uses a two cycle iterative approach for parameter estimation in such a large dataset. Each iteration consists of two distinct cycles, both following an EM algorithm approach. This iterative process will continue until convergence is achieved. We utilized a dataset from the National Alzheimer Coordinating Center (NACC) to estimate underlying measures of cognition based on a battery of observed neuropsychological tests. We assess the goodness of fit and the precision of the dynamic factor model estimators and compare it with a non-dynamic version in which temporal information is not used. The dynamic factor model is superior to a non-dynamic version with respect to fit statistics shown in simulation experiments. Moreover, it has increased power to detect differences in the rate of decline for a given sample size.

## Introduction

An increasing number of studies provide data on many variables across a large number of individuals in a longitudinal setting. These studies give us an unprecedented opportunity for epidemiological research on psychological measures over time and between subpopulations. For example, many observational and clinical trial studies of cognitive aging use neuropsychological test batteries to assess overall cognition and its specific domains [1]. Statistical tools have been developed to extract information from these evaluation tests in order to estimate a single or multiple latent cognitive indices. A common method for the estimation of such latent variables is confirmatory factor analysis (CFM) [2,3]. The repeated nature of the studies is often ignored in these models, even though recent studies attempt to capture the temporal information in order to increase performance of measures for cognitive change [4–6]. In psychology, the problem of estimating latent structures from repeated measurements has been addressed by the use of dynamic factor models (DFM). ere is a long literature on estimation theory and applications of DFMs, either direct autoregressive factor models (DAFS) or white noise factor score models (WNFS) [7–10]. These methods have been applied in studies with a relatively long time series (at least 70 repeated observations per subject) of a single or a small number of individuals [11–13]. On the other hand, in longitudinal epidemiological studies, we often have a large number of subjects with a very short time series, usually non-stationary, with typically 2 to 5 repeated observations per subject. Furthermore, in order to compare latent variables across subpopulation of interest cross-sectionally and over time, we require that the estimated factor scores are comparable across individuals. This implies certain restrictions on the factor model. Molenaar et al. (1992) extended DFMs to non-stationary time series [14]. Although models for large number of individuals and short time series are theoretically feasible by applying standard multivariate time series methods, they are computationally restrictive. As the number of individuals becomes large, so does the number of parameters to be estimated, and direct optimisation becomes harder and more time consuming. Markov Chain Monte Carlo methods for parameter estimation have been implemented [15,16], but these methods require long time series, typically in the hundreds of observations over time. On the other hand, Zuur et al. (2003) showed that parameter estimation using the EM (Expectation-Maximazation) algorithm in a relatively short time window (30 repeated observations) for 12 cross-sectional units can be easily implemented [17]. This methodological approach has its own limitations since applying the EM algorithm to a larger number of individuals and even shorter time series would substantially increase computation time [18]. The limitations of the data and complications of the estimation methods have resulted in a very narrow interest for DFM applications in large epidemiological studies.

In this paper, we provide statistical tools for analyzing DFMs using data which are typical in epidemiological studies with large number of participants and short non-stationary time series. Specifically, we develop an estimation algorithm, extending the classic EM algorithm, by developing an iterative two-cycle estimation process, following the steps of the ECME (Expectation/Conditional Maximization Either) algorithm [19]. This estimation method is flexible enough to be applicable in studies with multiple individuals, and short unequally spaced temporal information.

We apply the dynamic factor model to a variety of neuropsychological tests using data from the National Alzheimers Coordinating Center (NACC) study and estimate a smooth cognitive measure for each individual’s total cognition as well as measures for specific cognitive domains, such as memory, attention and language. We hypothesize that by incorporating longitudinal information into the factor models we increase the accuracy of the estimates of change over time and consequently increase power to detect differences between groups. We focus on a case-control sample using data where participants are selected to be cognitively normal. Cases in this study include participants that will convert to Mild Cognitive Impairment (MCI) after the period used in the analysis, while controls will continue having normal cognition for the next two follow-up visits after the end of the analytic period.

In the next section we describe the dynamic factor model and its estimation method. In §3, we assess its performance in estimating the underlying cognition and its domains and compare it with a factor model which ignores any longitudinal information (non-dynamic model). We apply the dynamic and non-dynamic versions of the factor model to clinical data collected by NACC, and compare their power of detecting differences in the rate of cognitive change for various sample sizes. Finally in §4, we conclude with a discussion of the methods and results including limitations and directions for future studies.

## Methods

In this section we describe the dynamic version of the factor model and its estimation process. The difference of the dynamic factor model for panel data from the non-dynamic version is that the former captures not only correlations between input variables but also autocorrelations and cross correlations of these variables of interest.

### Model

#### 

We let Ut denote the *nq* × 1 vector containing the unobserved cognitive indices of *q* factors for *n* subjects (with *q* < *n*) at time t, with t = 1,…, T. We assume that the dynamic properties of Ut can be captured by a Markov process. For illustration purposes, and without loss of generality, we first present the case where we have equally spaced observations and equal number of neuropsychological tests for each subject. We subsequently present the model for the general case with unequally spaced or missing observations. Hence, we form the following linear Gaussian state space model:

(2.1) $$y_{t}=BU_{t}+e_{t},\;\;\;e_{t}\sim N(0,D),$$

(2.2) $$U_{t+1}=TU_{t}+\eta_{t},\;\;\;\eta_{t}\sim N(0,I_{n}),$$

where B is the matrix of factor loadings with dimensions *np*×*nq*, with *p* denoting the number of observed variables, *y_t_* is a *np*×1 vector of observed neuropsychological measures per individual, T is *np*×*nq* transition matrix and *I_n_* is a *nq*×*nq* identity matrix and *e_t_* and *η_t_* are error terms [20,21]. The state space formulation described in (2.1) and (2.2) models the behavior of the unobserved state vector Ut over time using the observed values y1,….., yn. The state vector Ut is assumed to be independent of the error terms *e_t_* and *η_t_* for all t. In addition, the error terms *e_t_* and *η_t_* are assumed to be independent, identically distributed (i.i.d.) [22,23]. In general, the model defined by equations (2.1) and (2.2) is not identifiable. Zirogiannis and Tripodis (2014) state the conditions for identifiability for a general dynamic factor model [24]. In order for the model in (2.1) and (2.2) to be identifiable we must impose a certain structure. We first assume that the unobserved cognitive indices follow a multivariate random walk, so that *T* = *I_n_*. This is a reasonable assumption when modeling cognition for an aging population where the spacing of the observation period is roughly annual. Similar non-stationary models for psychological constructs have been suggested by Molenaar and Campbell (2009) and used, among others, by Hekler et al. (2013) and Gu et al. (2014) [12,25,26]. We also impose a structure on the factor loading matrix B and the variance of the idiosyncratic errors D. We assume that the factor loadings for each observed variable are the same for each individual in the study. This assumption is necessary in order to have comparable estimated cognitive indices across individuals. We also assume that participants in the study are conditionally independent and that the variance of the idiosyncratic errors is the same for all individuals. These assumptions result in a block diagonal structure for D. The imposed structure results in a model that is fully identifiable. The model can be easily extended for cases where non-diagonal block elements of B and D are not zero. The choice of the structure of B and D is specific to our application of interest.

##### Unequally spaced and missing observations

It is very common in longitudinal observational studies to have unequally spaced or missing observations. Let *τ_it_* be the distance between observations *t* and *t* + *τ_it_* of the *i^th^* subject, and *τ_t_* the vector with the distances between two subsequent observations at time *t*. Then we can re-write the state-space form of the multivariate random walks as:

$$\begin{array}{ll} y_{t}=BU_{t}+e_{t}, & e_{it}\sim N(0,D)\\ U_{t+\tau }=U_{t}+\eta_{t}, & \eta_{t}\sim N(0,Q_{t}) \end{array}$$

where $Q_{t}=\sum_{i=1}^{q}E_{i}\tau_{t}e_{i}$ with *E_i_* a *q*×*q* matrix with 1 for the element (i, i) and 0 everywhere else and *e_i_* is a 1×*q* vector with 1 for the element *i* and 0 everywhere else. This time-varying model can be used for unequally spaced and missing observations, as well as for forecasting for any *τ_n_* steps ahead.

### 2-step modified ECME Algorithm

The high dimensionality of the data vector *y_t_* makes estimation of our model rather problematic. Moreover, in biomedical applications such as the one we explore in this paper, we deal with cases where T is very small while n is very large. Usual Newton-type gradient methods do not work in this situation creating the need for a novel estimation approach. We introduce a modified ECME algorithm that makes estimation of the model specified in (2.1) and (2.2), feasible through an iterative two-cycle process. The 2-cycle modified ECME algorithm is an extension of the ECME algorithm developed by Liu and Rubin (1998), which itself is an extension of the widely known EM algorithm [27]. The modified ECME algorithm starts by partitioning the vector of unknown parameters Ψ into (Ψ_1_, Ψ_2_) where Ψ_1_ contains the elements of D that need to be estimated, while Ψ_2_ contains the relevant elements of B. We use the term “cycle” as an intermediary between a “step” and an “iteration” as in Meng and Dyk (1997) [28]. In the case of our modified ECME algorithm, every iteration is comprised of two cycles. Each cycle includes one E-step and one M-step, where the first cycle estimates Ψ_1_ and Ψ_2_ given the estimates of Ψ of the previous *iteration*, while the second cycle estimates Ψ_2_ given the estimates of Ψof the previous *cycle*.

The functional form of the complete-data log-likelihood at time period t is [29]:

$$\log\ell (\Psi )=\frac{1}{2}\log\left\{|D^{-1}|\right\}-\frac{1}{2}\left\{{\left(y_{t}-Bu_{t}\right)}^{\prime }\;D^{-1}\;(y_{t}-Bu_{t})-{\left(u_{t+1}-u_{t}\right)}^{\prime }\;(u_{t+1}-u_{t})\right\}$$

Since *u_t_* is unobserved, we can consider it missing and use the EM algorithm framework. In order to find the MLE, we need to calculate the distribution of the latent variable ut conditional on the observed values of *y_t_*. There is a long literature describing the EM procedure for factor analysis in cross-sectional data starting with Rubin and Thayer (1982) [30]. Applying the EM framework for longitudinal data we need to condition not only on the concurrent observed value of *y_t_* but on all the previous observed history *y*_1_, …, *y_t_*. As we will see in the following two subsections, we use the first cycle to obtain estimates for *u_t_* by conditioning on the concurrent observed variables, *y_t_*, and the second to update these estimates by conditioning on the history of the observed variable, *y*_1_, …, *y_t_* using the Kalman filter [31]. This iterative process will continue until the likelihood function stops increasing and convergence is achieved.

#### First cycle

During the *k^th^* iteration of the first cycle, the E-step of the 2-cycle ECME algorithm is:

(2.3) $$Z_{\Psi }(\Psi_{1},\Psi_{2};{\Psi_{1}}^{\left(k-1\right)},{\Psi_{2}}^{\left(k-1\right)})=E_{\Psi }\;\left\{\sum_{t=1}^{T}\log\ell_{t}\;\left[(\Psi_{1},\Psi_{2})|y_{t},\Psi_{1}^{\left(k-1\right)},\Psi_{2}^{\left(k-1\right)}\right]\right\}.$$

Following the notation presented in [29], the sufficient statistics are calculated in the (*k* − 1) iteration by the following equations:

(2.4) $$\begin{array}{l} \gamma^{\left(k-1\right)}={\left(B^{\left(k-1\right)}B^{\left(k-1\right)}+D^{\left(k-1\right)}\right)}^{-1}\;B^{\left(k-1\right)}\\ \omega^{\left(k-1\right)}=I-{\gamma^{\left(k-1\right)}}^{\prime }B^{\left(k-1\right)} \end{array}$$

The first M-step involves differentiating *Z*_Ψ_(Ψ_1_, Ψ_2_, Ψ_1_^(^*^k^* ^− 1)^, Ψ_2_^(^*^k^* ^− 1)^) with respect to Ψ_1_ and Ψ_2_ in order to obtain $\Psi_{1}^{\left(k\right)}$ and $\Psi_{2}^{\left(k/2\right)}$:

(2.5) $$Z_{\Psi }(\Psi_{1}^{\left(k\right)},\Psi_{2}^{\left(k/2\right)};{\Psi_{1}}^{\left(k-1\right)},{\Psi_{2}}^{\left(k-1\right)})\geq Z_{\Psi }(\Psi_{1},\Psi_{2};{\Psi_{1}}^{\left(k-1\right)},{\Psi_{2}}^{\left(k-1\right)}),$$

The first-cycle M-step is identical to the M-step of the traditional EM algorithm for factor analysis models [32]:

(2.6) $$B^{\left(k/2\right)}=C_{yy}{\gamma^{\left(k-1\right)}}^{\prime }{\left\{{\gamma^{\left(k-1\right)}}^{\prime }C_{yy}\gamma^{\left(k-1\right)}+n\omega^{\left(k-1\right)}\right\}}^{-1},$$

(2.7) $$D^{\left(k\right)}=n^{-1}\mathrm{diag}\left\{C_{yy}-C_{yy}\gamma^{\left(k-1\right)}B^{\prime }\right\},$$

Where *C_yy_* is the sample unconditional covariance matrix of $Y_{T}={\left(y_{1}^{\prime },\ldots ,y_{T}^{\prime }\right)}^{\prime }$, i.e. $E(Y_{T}Y_{T}^{\prime })=C_{yy}$. At the end of the first cycle we have updated estimates for all the elements of the variance matrix of the idiosyncratic errors, D, and intermediate estimates for the matrix of factor loadings, B. We use these estimates in the second cycle to get updated estimates for the factor loadings.

#### Second cycle

In the E-step of the second cycle we estimate $\Psi_{2}^{\left(k\right)}$. We proceed by calculating:

(2.8) $$Z_{\Psi_{2}}(\Psi_{2};\Psi_{1}^{\left(k\right)},\Psi_{2}^{\left(k/2\right)})=E_{\Psi_{2}}\left\{\sum_{t=1}^{T}\ell [\Psi_{2}|Y_{t-1},\Psi_{1}^{\left(k-1\right)},\Psi_{2}^{\left(k/2\right)}]\right\}|.$$

The second E-step involves forming the expected complete-data log likelihood conditional on *Y_t_*_−1,_ which is the set of past observations *y*_1,…,_*y_t_*_−1._ The subsequent M-step involves differentiating $Z_{\Psi_{2}}(\Psi_{2};\Psi_{1}^{\left(k\right)},\Psi_{2}^{\left(k/2\right)})$ with respect to Ψ_2_. We choose $\Psi_{2}^{\left(k\right)}$ such that:

(2.9) $$Z_{\Psi_{2}}(\Psi_{2};\Psi_{1}^{\left(k\right)},\Psi_{2}^{\left(k/2\right)})\geq Z_{\Psi_{2}}(\Psi_{2};\Psi_{1}^{\left(k\right)},\Psi_{2}^{\left(k/2\right)}).$$

Upon maximization of *Z*_Ψ_2__, the estimate $\Psi_{2}^{\left(k\right)}$ is used in the E-step of the first cycle of the next iteration. We calculate and maximize $Z_{\Psi_{2}}(\Psi_{2};\Psi_{1}^{\left(k\right)},\Psi_{2}^{\left(k/2\right)})$ by using the prediction error decomposition of the conditional likelihood [33]:

(2.10) $$\log\ell_{t}\;(\Psi_{2})=\log\frac{1}{2\pi }-\frac{1}{2}[\log|F_{t}|+|\upsilon_{t}^{\prime }F_{t}^{-1}\upsilon |],$$

Where *υ_t_* is the prediction error conditional on past history and *F_t_* is its variance. Quantities, *υ_t_* and *F_t_* can be estimated with the use of the Kalman filter, which is a set of recursions which allow information about the system to be updated every time an additional observation *Y_t_* is introduced [21]. Once *υ_t_* and *F_t_* are calculated, (2.10) is maximized with respect to Ψ_2_, as illustrated in (2.9).

## Results

In the next section, we assess and apply the model and the estimation process described in §2. We first assess the performance of the 2-cycle ECME estimator using a simulation study. We then apply the model in data from the NACC study. We also compare the dynamic factor model with a non-dynamic version in which temporal information is not used. The non-dynamic version is observationally equivalent to a Confirmatory Factor Analysis (CFM) model and it is defined solely by the observation equation (2.1). Estimation was done using a code written by the authors in Ox *Programming Language* [34].

### Simulation

The model from which we simulate is a variant used by Doz et al. (2011) which is based on a simulation scheme used by Stock and Watson (2002) [35,36]. We define:

(3.1) $$B=\left(\begin{array}{cccc} f & 0 & \cdots & 0\\ 0 & f & \cdots & 0\\ \vdots & \cdots & \ddots & \vdots \\ 0 & \cdots & \cdots & f \end{array}\right),\;\;\;D=\left(\begin{array}{cccc} d & 0 & \cdots & 0\\ 0 & d & \cdots & 0\\ \vdots & \cdots & \ddots & \vdots \\ 0 & \cdots & \cdots & d \end{array}\right),$$

With

1. f a *p*×1 vector of factors loadings with f_[K]_ ~ U(0,1) subject to $\sum_{k=1}^{p}f_{\left[K\right]}=1$
2. d a *p×p* diagonal matrix of variances for the idiosyncratic elements, with $d_{\left[k][k\right]}=f_{\left[k\right]}\frac{\beta_{k}}{1-\beta_{k}}$ with *β_k_* ~ *u*(0.1, 0:9)

where *k* = 1,…, *p*. We generate 1000 replicates from the model defined by (2.1), (2.2) and (3.1) with U_0_ ~ N(0, I*_n_*), for different combinations of sizes for observed tests, p, number of subjects n, and time points, T. Specifically, we use p = 5,10,15, n = 10,50,100,200,300 and T = 3,5,7,10,15.

The choice of these values corresponds to our specific application. We specify factor loadings which are the same across individuals who do not share any familial or other relationship. The coefficient *β_k_* is the ratio between the variance of the idiosyncratic component, e_t_, and the total variance of the corresponding observed variable, Y*_t_*. In the simulation, this ratio is drawn from a uniform distribution between the interval of (0.1, 0.9). This interval was chosen in order to avoid parameters at the boundary of the parameter space.

Estimation was done using the 2-cycle modified ECME and yielded estimates of the factor Û = (Û_1_, Û_2,…,_ Û*_T_*). Performance was measured by the trace statistic:

$$\frac{tr(U^{\prime }\hat{U}{\left({\hat{U}}^{\prime }\hat{U}\right)}^{-1}{\hat{U}}^{\prime }U)}{tr(U^{\prime }U)}.$$

The trace statistic is a multivariate version of the R^2^ of the regression of the true factors on the estimated factors [35]. A number close to 1 implies a good approximation of the estimated latent variable to the true factor. We used the trace statistic, *TR_DFM_*, as a performance measure for the

Dynamic factor model. We also obtained estimates of the latent factor using a non-dynamic factor model defined only by equation (2.1). In order to have comparable results, we also used the 2-cycle modified ECME for the non-dynamic version of the factor model. We then calculated the equivalent trace statistic, *TRCFM* for the non-dynamic model. We use the ratio of the two trace statistics, $\frac{{TR}_{\mathrm{DFM}}}{{TR}_{\mathrm{CFM}}}$, as a comparison measure of the two models. Values above 1 imply that the dynamic model has superior performance to the non-dynamic version, with respect to how close the estimated and the true factors are.

Table 1 reports the results of the trace statistics from the simulation experiment. The numbers in the table refer to the average across 1000 replicates. As an example, we use the case of T = 3, n = 300, and p = 5; 86% of the variability of the true, simulated factor is explained by the factor estimated by the DFM. The explained variability using DFM is 1% higher than the explained variability using CFM. The goodness of fit of the estimated factors, as measured with *TR_DFM_*, increases with the size of individuals n in the sample, and the number of repeated observations per individual T. *TR_DFM_* varies from 0.77 for a small-n, and small-T sample to 0.97 for a moderate n-moderate T. The goodness of fit of the estimators does not improve as the number of observed tests p per individual increases for a given size n and T. Moreover, the dynamic factor model always performs at least as good as the non-dynamic version. The relative performance of the dynamic factor model increases with T. Based on $\frac{{TR}_{\mathrm{DFM}}}{{TR}_{\mathrm{CFM}}}$, the relative performance of the dynamic model ranges from 1% better goodness of fit compared to the non-dynamic version when n = 10, p = 5 and T = 3 to 9% when n = 300, p = 15 and T = 15.

### Application

Alzheimers Disease (AD), the most common form of dementia, is a significant cause of disability and mortality among the elderly. The latest figures show that 5.2 million people in the US, approximately 14% of the population over age 70, are afflicted by AD [37]. As the population ages over the next several decades, this number is expected to increase [38]. The only definitive way to diagnose AD is post-mortem, but neuropsychiatrists reach a pre-mortem diagnosis by reviewing and discussing the subject’s clinical history, as well as scores from a variety of neuropsychological evaluation tests [39]. The results of the neuropsychological tests which are part of the batteries can exhibit high within-subject variability [40] and may make diagnosis difficult. Moreover, the emphasis in Alzheimers disease clinical research has shifted to developing interventions before symptoms onset. In order to address this need, researchers are required to develop cognitive measures which discriminate between cognitively healthy subjects and individuals with small cognitive changes who will convert to mild cognitive impairment (MCI).

We used the NACC dataset with visits from September 2005 to June 2013 for testing and evaluation. NACC serves as a repository for data collected at 34 past and present Alzheimer’s Disease Centers (ADCs) throughout the United States. The ADCs conduct clinical and biomedical research on Alzheimer’s disease and related disorders. Centers enroll their study subjects in various ways, including referral from clinicians, self-referral by patients themselves or concerned family members, active recruitment through community organizations, and volunteers who wish to contribute to research. Most centers also enroll volunteer control subjects. Study subjects at each center are best regarded as a case series, not necessarily representing all cases of disease in a defined population. More information on the study can be found in Morris et al. (2006) [41].

**Figure 1:**
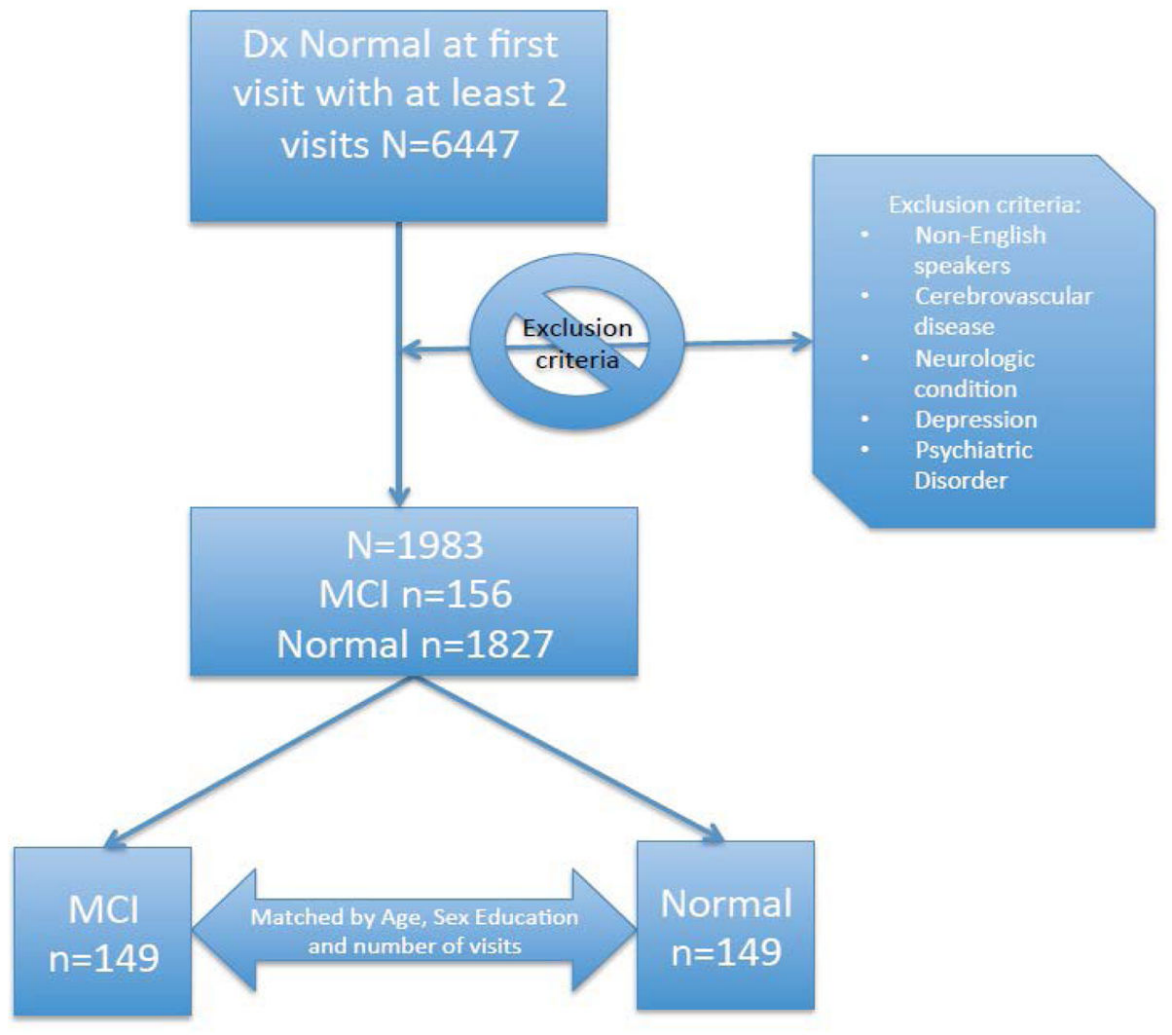
Description of analytic sample

We focus on a study sub-sample which includes cognitively healthy participants at initial visit. For all subjects we only considered their neuropsychological test results while cognitively healthy, even though some converted to MCI state at a later visit. For those participants who did not convert to MCI during our observation period, we only considered those with at least 4 visits. To avoid the risk of healthy participants converting to MCI at a future visit, we restricted our analytic period by excluding from further analyses the last two measurement occasions for that group. Consequently, normal controls remain cognitively normal for at least 2 years after the end of the analytic period used in this study. For those participants who converted to MCI we considered those with at least 1 follow-up with normal cognition. We also excluded non-English speakers as well as subjects with a number of comorbidities: history of stroke, history of transient ischemic attack, history of other cerebrovascular diseases, Parkinson’s disease or other Parkinsonism disorder, history of seizures, history of any brain trauma or other neurologic conditions, history of depression or psychiatric disorder. We then created two balanced groups, with n = 149 each, matched by age, sex and education which differ only in their future cognitive state: one group converts to MCI at the following visit after the end of the analytic period (converters), while the other group remains cognitively normal for at least the next two subsequent visits (non-converters) beyond the end of the restricted observational period. The description of the sample is given in figure 1. The mean (SD) age at initial visit is 75.7 (7.5) with 15.4 (2.5) average years of education. There are 170 (57.1%) women in the sample with 3.0 (1.2) visits on average, and 2.3 (1.3) years of follow-up since the initial visit.

We considered four factor models using different neuropsychological measures according to their relation to a specific domain: i) memory, ii) attention-psychomotor speed, iii) language and iv) general cognition. For each factor model, we run both a dynamic and a non-dynamic version. We estimated all models using a MacBook Pro with a Intel Core i7 2.3GHz processor on OS X Yosemite. Estimation times vary from 12.01 second for the language domain, to 46.97 seconds for the attention-psychomotor speed domain. For the general cognition factor, which includes all 11 neuropsychological scores, estimation time was 25.31 minutes. For both versions of the model, the one step-ahead prediction errors were tested for normality and residual autocorrelation. Even though both the dynamic and the non-dynamic version of all four factor models indicated non-normal errors (e.g. for general cognition, p-value<0.0001 for Bowman-Shenton test for normality and for Box-Ljung portmanteau test for autocorrelation at lag 1), further investigation showed that this is caused by outliers from seven participants. These participants have significantly lower estimated factors at the last visit, which may indicate misdiagnosis or untimely diagnosis of MCI. For each neuropsychological test, we run mixed effects regressions using PROC MIXED in SAS 9.3 with random intercepts and random slopes for time to test the hypothesis that there are significant differences in the rate of change by group (converters vs non-converters). We also used mixed effects regression on the factor scores estimated by the dynamic as well as the non-dynamic factor models. Table 2 shows the factor loadings for each domain and table 3 shows the estimated annual rate of change for each of the neuropsychological tests and for the non-dynamic and dynamic factors. For ease of comparison, all outcomes have been standardized, using the mean and standard deviation of all cognitively healthy NACC participants. We note that there is no significant annual change for the group of non-converters for all neuropsychological measures, with the exception of logical memory: delayed, and for the estimated factors. For the converters, only MMSE, Trails B and Verbal Fluency Test: vegetables show significant decrease at the 5% level, while the factors from the simple (non-dynamic) factor model for attention and language show significant decrease over time. For the dynamic factor model estimates, all three domains and total cognition estimates show significant decreases over time for the group which progressed to MCI at the next follow-up period. For the non-dynamic factor model estimates however, the total cognition factor and the memory factor do not show any difference. Given that an important feature that leads to an MCI diagnosis is manifestation of significant cognitive decline, it is important to note that the dynamic factor model estimates show evidence of decline even before conversion to MCI. We also note that both the dynamic and the non-dynamic version of factor models show significant differences in the annual rate of change between groups. In general, the estimates of difference of the Factor models are larger and have lower p-values than the estimates of input variables. Furthermore, the estimates of difference from the dynamic factor model are at least as high with larger p-values as the equivalent estimates of the simple factor models. This difference is due to the fact that DFM incorporates the longitudinal aspect of the psychometric results of every patient. This may be an indication of increased power for the dynamic factor model, which we explore in the next sub-section.

#### Power analysis

We also investigated the performance of the observed indicators and the estimated factors with respect to power. Our main aim remains the detection of differences by group in the rate of change. In order to assess the power of each outcome, we follow a bootstrapping scheme using the data described in the previous section. We first assume that there is, indeed, a difference in the annual rate of change between normal controls and MCI while they are both cognitively normal. For a given sample size n, we perform the following steps:

##### Simulation scheme

1. Select n/2 matched pairs with replacement.
2. Estimate factor scores for all domains and for total cognition using simple and dynamic factor models.
3. Run a mixed effect regression on the estimated factor using time since first visit, group (converters Vs non-converters) and time×group interaction, along with age at initial visit as covariates.
4. Is the estimate of time×group interaction significant at the 5% error level?
5. Repeat for 1000 times.

Table 4 shows the power of detecting significant differences for different sample sizes for all outcomes. We note that power of the dynamic factor model estimates is higher than the power of the non-dynamic version. For the total summary index, the power for the dynamic version varies from 49.9% for n = 120 to 96.7% for n = 240. The power for the non-dynamic version is much lower and goes up to 76.9% for n = 240. These results indicate that a smaller sample size is required for a given power in order to find significant differences in the rate of change by groups. Using the results from table 4, we can calculate the required sample size for both the dynamic and the non-dynamic factor models for an 80% power at a = 5%. For the total cognition index, the DFM model requires a sample size of 187 while the non-dynamic version (CFM) requires a sample size of 252. We get similar results for the other domains: memory (*n*_DFM_ = 370, *n*_CFM_ = 485), attention (*n*_DFM_ = 334, *n*_CFM_ = 546), language (*n*_DFM_ = 414, *n*_CFM_ = 1419).

We also note that the power of the factor models is always higher than the power of the individual neuropsychological tests. This indicates that using factor models increases the power of detecting significant differences in the rate of change. One notable exception is the Boston Naming Test (BNT) in the language domain. BNT has a higher power for all sample sizes considered compared to the non-dynamic factor estimates. It also has a higher power than the dynamic factor model when n = 120. For larger sample sizes, the dynamic factor model estimates have higher power than BNT.

## Conclusion

In this article, we developed an algorithm to estimate a dynamic factor model for data typical in large epidemiological studies and apply it on latent cognitive variables. We compared it with equivalent factor models which do not use temporal information in the estimation, and showed that the dynamic factor model estimates are more accurate as reflected by comparison of fit statistics in simulation experiments. They are also more precise than the non-dynamic version estimates as shown by improved power to detect differences in the rate of decline. Since the estimated latent index is a weighted average of the concurrent observed values, the reason for the improved performance of the dynamic factor model is due to the fact that the weighting scheme of DFM takes into account any within-subject variability over time and any cross-correlation of tests. In the non-dynamic version, weights depend on the correlation between tests as well as on between-subject variability. Measures that are highly correlated or have increased between-subject variability will receive higher weight. The main limitations of the non-dynamic approach are that we do not use any information from the within-subject variability over time. If we do not account for variability over time we may over (under) inflate the weights. In the dynamic factor model, the estimated latent variable is a weighted average of observed values from all time points. Concurrent values are weighted higher than observations further back into the past which will be discounted exponentially. The rate of discount will depend on the variability of each observed measure over time. For example, in the dynamic factor model, past observations of measures that are stable over time will be discounted less. Koopman and Harvey (2003) provide a general description of the weighting schemes for the model defined by equations (2.1) and (2.2) [42].

The dynamic factor model can be extended to allow for observed variables loading to multiple factors or for studies where participants may be clustered due to familial or other relationship. The current model is applicable to data with short temporal component and unequally spaced observations. This is a particular strength of the estimation algorithm, since most of the observational studies on cognition have these specific characteristics. A limitation of the current study is that the estimated factor is not validated with changes in biomarkers, such as volumetric data from MRI scans. Additionally, even though NACC battery is well validated and we consider the tests which load to specific domains as known, this may not be true in other applications. Another limitation of this current study is the use of aggregate scores for each test rather than the scores of each specific item used in each test. Crane et al. (2008) show that it is advantageous to use the item scores to derive latent factors in longitudinal studies [43]. Unfortunately, in the NACC study, as in many large studies, the data for items are not readily available for all participants. The methodology presented in this paper can be easily applied in most large studies where only the aggregate scores for each test are available. The dynamic factor model is particularly useful when we are interested in finding differences in the rate of cognitive change between groups. This advantage can be used in future observational studies researching the heterogeneity in rates of progression of MCI and AD patients. The DFM model can be easily used in searching specific thresholds above which the risk of conversion to MCI increases. This is especially important for designing future clinical trials that need to identify healthy participants at high risk of significant decline in cognition.

## Figures and Tables

**Table 1:** Performance of factor estimators from 1000 simulations. Trace statistics for various time lengths and sample sizes.

		*T* =3		*T* =5		*T* =7		*T* =10		*T* =15	
		*TR_DFM_*	$\frac{{TR}_{\mathrm{DFM}}}{{TR}_{\mathrm{CFM}}}$	*TR_DFM_*	$\frac{{TR}_{\mathrm{DFM}}}{{TR}_{\mathrm{CFM}}}$	*TR_DFM_*	$\frac{{TR}_{\mathrm{DFM}}}{{TR}_{\mathrm{CFM}}}$	*TR_DFM_*	$\frac{{TR}_{\mathrm{DFM}}}{{TR}_{\mathrm{CFM}}}$	*TR_DFM_*	$\frac{{TR}_{\mathrm{DFM}}}{{TR}_{\mathrm{CFM}}}$
n = 10	P = 5	0.77	1.00	0.80	1.02	0.83	1.02	0.86	1.02	0.88	1.01
	P = 10	0.77	1.00	0.78	1.02	0.81	1.03	0.83	1.03	0.86	1.02
	P = 15	0.77	1.00	0.77	1.02	0.78	1.03	0.81	1.03	0.84	1.02
n = 50	P = 5	0.85	1.01	0.87	1.02	0.90	1.02	0.92	1.02	0.93	1.01
	P = 10	0.85	1.01	0.86	1.02	0.87	1.03	0.90	1.03	0.92	1.02
	P = 15	0.85	1.01	0.85	1.02	0.86	1.03	0.88	1.03	0.91	1.02
n = 100	P = 5	0.86	1.01	0.89	1.02	0.91	1.02	0.93	1.02	0.95	1.01
	P = 10	0.86	1.01	0.86	1.02	0.89	1.03	0.92	1.03	0.94	1.02
	P = 15	0.87	1.01	0.86	1.02	0.88	1.03	0.90	1.03	0.93	1.03
n = 200	P = 5	0.88	1.01	0.90	1.02	0.92	1.02	0.94	1.02	0.96	1.02
	P = 10	0.87	1.01	0.89	1.02	0.91	1.03	0.93	1.03	0.95	1.02
	P = 15	0.89	1.01	0.88	1.02	0.89	1.03	0.92	1.04	0.93	1.03
n = 300	P = 5	0.86	1.01	0.91	1.02	0.93	1.03	0.95	1.05	0.96	1.03
	P = 10	0.87	1.01	0.90	1.02	0.91	1.03	0.94	1.03	0.97	1.07
	P = 15	0.90	1.01	0.89	1.02	0.91	1.03	0.93	1.04	0.96	1.09

**Table 2:** Factor loadings for each domain

Test	Memory	Attention	Language	Total
MMSE	0.22			0.15
Logical Memory: Immediate	0.34			0.08
Logical Memory: Delayed	0.34			0.05
Digits Forward		0.25		0.06
Digits Backward		0.27		0.12
WAIS		0.16		0.07
TRAILS A		0.20		0.08
TRAILS B		0.23		0.08
Animals			0.35	0.14
Vegetables			0.33	0.08
Boston Naming Test			0.28	0.12

**Table 3:** Parameter estimates for annual rate of change for all neuropsychological tests

Domain	Test	Did not progress to MCI		Progress to MCI		Difference	
		Estimate (SE)	p-value	Estimate (SE)	p-value	Estimate (SE)	p-value
Memory	MMSE	−0.02 (0.03)	0.390	−0.06 (0.03)	0.027	0.04 (0.04)	0.343
	Logical Memory:	0.04	0.202	−0.04	0.140	0.08	0.052
	Immediate	(0.03)		(0.03)		(0.04)	
	Logical Memory:	0.06	0.035	−0.02	0.404	0.08	0.038
	Delayed	(0.03)		(0.03)		(0.04)	
	Factor CFM	0.04 (0.03)	0.202	−0.04 (0.03)	0.148	0.08 (0.04)	0.037
	Factor DFM	0.05 (0.04)	0.253	−0.09 (0.04)	0.026	0.14 (0.06)	0.020
Attention-Psychomotor Speed	Digits Forward	−0.04 (0.02)	0.127	−0.03 (0.02)	0.268	−0.01 (0.03)	0.770
	Digits Backward	0.01 (0.03)	0.680	−0.03 (0.03)	0.201	0.04 (0.04)	0.231
	WAIS	−0.03 (0.02)	0.073	−0.03 (0.02)	0.124	0.01 (0.02)	0.839
	TRAILS A	0.03 (0.03)	0.196	−0.01 (0.03)	0.599	0.05 (0.04)	0.199
	TRAILS B	0.01 (0.02)	0.742	−0.05 (0.02)	0.039	0.03 (0.03)	0.089
	Factor CFM	0.00 (0.02)	0.852	−0.04 (0.02)	0.001	0.04 (0.02)	0.048
	Factor DFM	−0.00 (0.03)	0.914	−0.08 (0.03)	0.003	0.04 (0.04)	0.046
Language	Animals	−0.00 (0.02)	0.847	−0.04 (0.02)	0.056	0.04 (0.03)	0.222
	Vegetables	−0.02 (0.02)	0.493	−0.05 (0.03)	0.029	0.04 (0.04)	0.285
	Boston Naming Test	0.04 (0.03)	0.100	−0.02 (0.03)	0.285	0.06	0.076
	Factor CFM	−0.00 (0.02)	0.916	−0.04 (0.02)	0.014	0.04 (0.03)	0.094
	Factor DFM	−0.02 (0.03)	0.515	−0.09 (0.03)	0.001	0.07 (0.04)	0.069
Total	Factor CFM	0.05 (0.02)	0.058	−0.04 (0.02)	0.098	0.09 (0.03)	0.012
	Factor DFM	0.01 (0.03)	0.654	−0.12 (0.03)	<.001	0.13 (0.05)	0.004

**Table 4:** Power analysis for group differences

Domain	Test	*n* = 120	*n* = 150	*n* = 180	*n* = 210	*n* = 240
Memory	MMSE	4.9	3	3.1	1.3	0.9
	Logical Memory: Immediate	15	20.3	23.1	25.2	29.8
	Logical Memory: Delayed	14.4	20.2	24.9	28.4	34.3
	*Factor-CFM*	17.2	22.2	26.8	32.2	38.1
	*Factor-DFM*	21.9	28.7	33.7	40.1	51
Attention-Speed	Digits Forward	2.3	0.9	1	0.4	0.4
	Digits Backward	4.4	4.1	3.9	1.8	1.9
	WAIS	1.7	0.7	0.2	0	0
	TRAILS A	12.6	14.8	16.1	18.8	16.6
	TRAILS B	12.5	13.4	17.1	15.8	16.6
	*Factor-CFM*	15.2	17.7	23.7	28.3	32.8
	*Factor-DFM*	21.5	24.4	30.6	45.9	52.4
Language	Animals	5.6	5.4	5.7	5.6	2.7
	Vegetables	5.8	4	4.4	3	1.4
	Boston Naming Test	19.1	20.8	24.5	23.4	22.1
	*Factor-CFM*	10.3	13.2	12.6	15.9	15.7
	*Factor-DFM*	14.8	20.8	31.7	37	37.1
Total	*Factor CFM*	39.3	49.2	59	65.5	76.9
	*Factor DFM*	49.9	66	81.1	92.8	96.7
